# Eye reactions under the influence of drugs of abuse as measured by smartphones: a controlled clinical study in healthy volunteers

**DOI:** 10.3389/fnins.2024.1492246

**Published:** 2025-01-07

**Authors:** Kiki W. K. Kuijpers, Karl Andersson, Maria Winkvist, Marieke Niesters, Monique van Velzen, Fred Nyberg, Albert Dahan, Markku D. Hämäläinen

**Affiliations:** ^1^Department of Anesthesiology, Leiden University Medical Center, Leiden, Netherlands; ^2^Skillsta Teknik Design och Kvalitet AB, Vänge, Sweden; ^3^IGP, Rudbeck Laboratory, Uppsala University, Uppsala, Sweden; ^4^Kontigo Care AB, Uppsala, Sweden; ^5^Department of Pharmaceutical Biosciences, Uppsala University, Uppsala, Sweden

**Keywords:** substance use disorder, pupillometry, opioids, central stimulants, benzodiazepines, cannabis

## Abstract

**Background:**

It is known that illicit and prescribed drugs impact pupil size, eye movement and function. Still, comprehensive quantitative evaluations under known ambient light conditions are lacking, when smartphones are used for monitoring.

**Methods:**

In this clinical study (NCT05731999), four medicinal products with addiction risks were administered to 48 subjects (18–70 years old, all with informed consent, 12 subjects per drug). Videos captured by smartphones at ~50 lux and ~ 500 lux documented the eye’s reaction before and after controlled intake of single doses of oral oxycodone (20 mg), lorazepam (2 mg), lisdexamphetamine (70 mg) and inhaled cannabis flos (65 mg with 22% THC) over a 5-h test period. Data from three observational tests, non-convergence (NC, ability to cross the eyes), nystagmus (NY), and pupillary light reflex (PLR) were converted into 24 key features that represent different eye characteristics.

**Results:**

Of the acquired data, 87–97% produced key features. At peak drug plasma concentration, oxycodone constricted pupils (*p* < 0.001); lorazepam induced non-convergence (*p* < 0.001); lisdexamphetamine induced dilated pupils (*p* < 0.001), irrespective of ambient light conditions. Inhaled cannabis induced miosis (*p* = 0.05 at ~50 lux, *p* = 0.10 at ~500 lux), a reduced light-induced amplitude (*p* = 0.003 at ~50 lux, *p* = 0.3 at ~500 lux) and redness of the sclerae (*p* = 0.14 at ~50 lux, *p* = 0.007 at ~500 lux). The drug effect lasted at least 5 h (*p* < 0.005) except for inhaled cannabis (2–3 h, *p* < 0.05).

**Conclusion:**

The ocular response to oxycodone, lorazepam, lisdexamphetamine and cannabis, as measured under controlled light conditions using a smartphone-based assessment, demonstrated distinct and readily distinguishable patterns for each substance.

**Clinical trial registration:**

Identifier, NTC05731999.

## Introduction

Substance Use Disorder (SUD) is a multifaceted condition characterized by the compulsive use of substances despite adverse consequences, profoundly impacting individuals’ lives (Koob and Volkow, 2016; Goode and Maren, 2019). This complex disorder is recognized as a brain disease by the American Psychiatric Association, associated with impaired control of drug use, social impairment, risk level use and factors related to tolerance and withdrawal (American Psychiatric Association DSM-5 Task Force, 2013). In addition, relapse is a common feature (American Psychiatric Association DSM-5 Task Force, 2013; Sliedrecht et al., 2019). Central to understanding SUD is the impact of repeated substance use on brain areas related to decision-making, impulse control, and reward processing (Koob and Volkow, 2016). Individuals with SUD often struggle to abstain from substance use despite experiencing significant adverse effects on physical health, social relationships, employment, and overall well-being (Koob and Volkow, 2016; American Psychiatric Association DSM-5 Task Force, 2013; Sliedrecht et al., 2019). Therefore, intensive long-term outpatient care with extensive monitoring has been proposed as a cost-effective option for managing substance use disorders (Treatment Center for Substance Abuse, 2006).

Historically, evaluating pupillary reactions to light, known as the pupillary light reflex (PLR), has been crucial for assessing neurological conditions. As a key indicator of brainstem function (Robert and Charlotte, 2018) within the central nervous system (CNS), PLR aids in diagnosing various neurological conditions such as concussions (Robert and Charlotte, 2018), optic nerve injury, oculomotor nerve damage, brainstem lesions, and the use of certain medications such as opioids or central stimulants (Mathôt, 2018; Knaggs et al., 2004; Holze et al., 2020). Additionally, deviations in eye-related responses, such as the lack of smooth pursuit and the onset of nystagmus, can also indicate CNS impairment (Strupp et al., 2021).

Patients with SUD often use drugs that significantly influence pupillary reactions. For instance, opioids are known to induce dose-dependent reductions in pupil size and constriction velocity in response to light stimuli (Knaggs et al., 2004). This effect is attributed to the activation of the pupillary sphincter muscle, resulting in miosis (Knaggs et al., 2004). Likewise, cocaine and amphetamines can cause pupil dilation due to their ability to increase norepinephrine levels (Holze et al., 2020). While benzodiazepines are believed to have minimal effects on pupillary responses, high doses can lead to eye-related features typical of CNS depressants, such as nystagmus (Drummer, 2002). Additionally, the cannabinoid THC is known to trigger horizontal gaze nystagmus (DeGregorio et al., 2021) and phencyclidine has been reported to initiate both vertical and horizontal nystagmus (Dominici et al., 2015).

Despite the potential utility of pupillary reactions in identifying drug use, distinguishing drug-related signals from individual variations and environmental factors remains challenging (Pickworth et al., 1997). Factors such as ambient light variations, near fixation, arousal, mental effort, and age can influence eye-related responses, complicating their interpretation in the context of drug detection (Mathôt, 2018; Guillon et al., 2016). Furthermore, attempts to use recorded eye reactions to identify drug use have yet to achieve widespread adoption due to concerns about signal variability and the impact of ambient light levels during measurements (Porath-Waller and Beirness, 2010; McKay and Larson, 2021; Monticelli et al., 2015; Kongsgaard and Høiseth, 2019; Campobasso et al., 2020). For instance, studies have shown that nystagmus can occur in drug-naive individuals with a frequency of 10–20%, further complicating drug detection specificity (DeGregorio et al., 2021; Campobasso et al., 2020).

In recent years, smartphones have emerged as powerful tools for various health applications, ranging from self-guided health information to vital sign measurement and disease management (Strand et al., 2023). Despite their widespread use, smartphones have not been widely utilized for characterizing eye reactions to detect illicit drug use, although this potential application has been proposed (Fazari, 2011). Some of the many challenges with smartphones include mastering an image taken with consumer grade cameras of varying quality in visible light where surrounding light sources cause reflexes. Previous smartphone applications have primarily focused on quantifying pupillary reactions, with mixed results regarding their clinical utility (Shin et al., 2016; McKay and Larson, 2021; McAnany et al., 2018). However, recent advancements in smartphone technology may address previous concerns related to measurement precision and quality (Sousa et al., 2021; Piaggio et al., 2021).

For alcohol use, a variety of long-term and short-term tests (Årving et al., 2021; Helander et al., 2012) based on plasma and urine as well and breathalyzers (Wallden et al., 2024) exist to support the care process. Today, electronic sensing devices linked to central computer servers are used to monitor alcohol sobriety (Wallden et al., 2024; Paprocki et al., 2022). Digitally connected breathalyzers that predict potential relapses by analyzing captured behavioral patterns (Zetterström et al., 2021) also exist. The landscape for detecting drug use remains fragmented and less developed, and daily recurrent substance use monitoring in intensive outpatient care is not available for subjects with SUD. While most drugs can be detected in blood and urine samples, obtaining such samples from patients can be challenging in outpatient settings and may complicate the therapeutic alliance (Kaye et al., 2014). Urine and saliva-based test panels offer quicker results and are suitable for facilities without laboratory resources, but there are currently no commercial drug sensors designed for at home use, limiting the therapists ability to monitor patient sobriety. The lack of available tools presents a substantial challenge in the effective support and management of patients with substance use disorder within outpatient care settings.

Our aim is to explore the feasibility of using smartphone technology to detect illicit drugs by utilizing commonly used therapeutic drugs. We try to utilize the widespread availability, sophisticated sensor and data processing capabilities of modern smartphones to overcome the limitations inherent in conventional drug detection methodologies. The present study is only one of the several parallel activities (Månflod et al., 2024) contributing to this broader effort.

## Materials and methods

### Study design and ethics

The study had an explorative, randomized, parallel open-label feasibility design that took place in a single center in the Netherlands. The study is reported in line with CONSORT pilot study guidelines (Eldridge et al., 2016) where the workflow is described below, with details in the Supplementary material. The main objectives of the study were to collect data with consumer grade electronics and compare the findings with previously published claims mainly made using specialized precision equipment found in hospitals. The study was conducted at the Anesthesia and Pain Research Unit of the department of Anesthesiology in Leiden University Medical Center (LUMC) between February and July 2023. The study protocol was approved by the ethics committee METC-LDD (Leiden, the Netherlands; approval date 02-Feb-2023). All study procedures were performed according to good clinical practice guidelines and adhered to the tenets of the Declaration of Helsinki. The study was compliant with ISO 14155 to ensure the scientific conduct of the clinical investigation and the credibility of the results. The study including study data was registered in the public trial register clinicaltrials.gov with identifier NCT05731999. Prior to study enrollment, all subjects provided written informed consent.

Eye characteristic data was collected with an app embedded in the Previct platform (version 2.18, Previct Drugs; Kontigo Care AB, Uppsala, Sweden) as previously described (Månflod et al., 2024). To capture the videos of both eyes, the participants received either a Samsung S22 (Samsung Electronics Co., Yeongtong-gu, Suwon, South Korea) or iPhone 13 mini (Apple Inc., Cupertino, CA, USA) smartphone. After an initial visit with brief training on how to use the Previct Drugs App, video-recordings of eye characteristics were done by the participants themselves both at home and during the visit when the drug was taken at the department of Anesthesiology at LUMC. Adverse events were recorded between study enrollment and the end of study for each subject and were assessed based on severity and relatedness to study procedures.

### Randomization

The purpose of randomization in this study was to ensure that each drug had representatives of different ages and different eye colors, to avoid bias in the assessment of eye response to the medicinal products. Participants (*n* = 48) were therefore randomized based on eye color [light (blue/green/gray) or dark (brown)] and age category (18–20 years, 50–70 years, or 18–70 years) into one of four treatment groups (12 participants in each group). Next, subjects visited the research unit after fasting for at least 6 h. During this visit, subjects were administered drugs in the form of one of four medicinal products: oxycodone (20 mg oxycodone HCl Teva, oral immediate release), lisdexamphetamine (70 mg Elvanse, oral), lorazepam (2.0 mg Lorazepam Aurobindo, oral) or medicinal cannabis [Bedrocan, Bedrocan International B.V., 65 mg with 22% THC (14.3 mg of which ~5 mg are bioavailable)]. Oxycodone, lisdexamphetamine and lorazepam were provided in tablet form. Medicinal cannabis was inhaled using the Volcano vaporized system as previously described (Bonfiglio et al., 2006), all other drugs were orally ingested using 100 mL of non-carbonated water. Administered doses followed a recommended high daily dose for the respective medical use of the medicinal products.

### Subjects

Healthy male and female volunteers, aged 18–70 years with body mass index 18.5–30 kg/m^2^ and weight 50–100 kg were included (Table 1 and Supplementary material). Women of the childbearing age group had to provide negative urine pregnancy test at enrollment, and prior to drug dosing. Additional inclusion criteria were: healthy based on medical history and physical examination at screening, no current drug use confirmed by negative urine drugs tests, and able to operate the Previct Drugs App without the App producing error messages after initial training. Exclusion criteria were: blind, deaf, pregnant or lactating, abnormal ECG at enrollment, current or previous alcohol abuse, current or history of a psychiatric disorder, current condition or treatment with medication that may affect eye measurements, or a known allergy to study drugs. Alcohol habits were evaluated using Alcohol Use Disorders Identification Test (AUDIT) and subjects were excluded if the AUDIT result was ≥6 points for women or ≥ 8 points for men. All subjects who exhibited signs of psychiatric disorders and/or addictions to other stimuli, as determined by the M.I.N.I. questionnaire, were excluded from the study.

**Table 1:** Description of the subjects in the clinical study.

	Group 1	Group 2	Group 3	Group 4
#Subjects	12	12	12	12
Male	4	2	5	3
Female	8	10	7	9
iPhone 13 mini	6	6	6	6
Samsung S20	6	6	6	6
Age*** range	19–69	19–57	19–70	19–60
Age median	21	21	20.5	21
Drug family	Opioids	Phenethylamines	Cannabinoids	Benzodiazepines
Drug used	Oxycodone	Lisdexamphetamine	Cannabis	Lorazepam
Dose	20 mg	70 mg *	65 mg **	2 mg
Route of administration	Oral	Oral	Inhalation	Oral

* 70 mg lisdexamphetamine is metabolized to approximately 20 mg dexamphetamine. ** The cannabis variety used contains 22% THC, and the calculated delivered bioavailable THC dose becomes 5 mg. ***Age in years.

### Data collection

**Figure 1:**
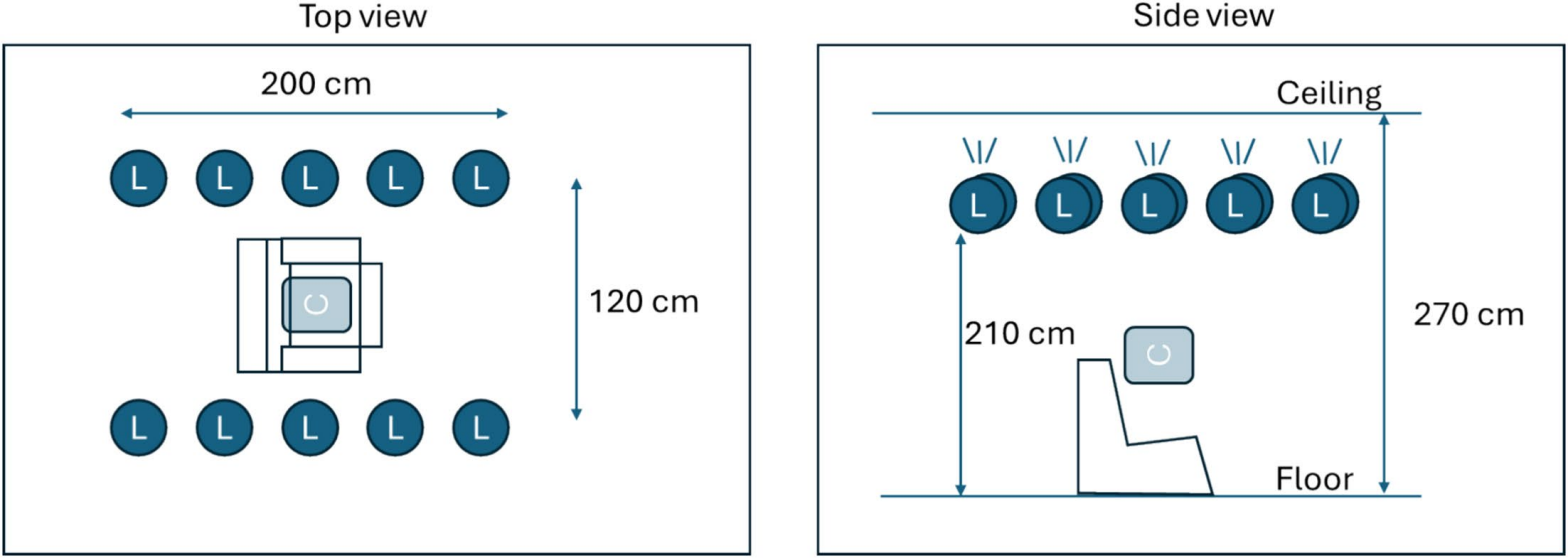
Description of the measurement area. Ten light sources (L) were mounted facing upwards near the ceiling. Light sources could be controlled in terms of intensity. Two fixed intensity levels were created, producing either 50 lux or 500 lux at calibration position C.

The Previct Drugs App was programmed to collect data using the default camera settings in the smartphone (30 frames per second, auto exposure, no calibrations required) from three different eye-scanning procedures: pupillary light reflex, non-convergence and horizontal nystagmus (Table 2). The minimal reporting guidelines, as outlined by Dunn et al. (2024), were followed. Pupillary light reflex data collection was made using the back camera to allow use of the flashlight of the phone, meaning that the subject had to flip the phone and position it centered at approximately 30 cm distance from the face through digitized voice guidance (Andersson et al., 2023). Non-convergence and horizontal nystagmus were collected using the front camera at approximately 25 cm distance, also with help of digitized voice guidance. Ambient light conditions were estimated by the app each time an eye-scanning procedure was conducted. A “test” refers to completing all these three procedures, which took approximately 5–10 min. The test was self-administered, i.e., the subject is making the test themselves while sitting or standing unless otherwise noted. The test was conducted within the designated area defined by the arrangement of light sources (Figure 1), typically but not exclusively positioned near the calibration point.

**Table 2:** Description of the eye-scanning procedures and the corresponding key features extracted from acquired data.

Procedure	Description	Key features	Description
Pupillary light reflex (PLR)	Use voice guidance to instruct the person to (1) flip the phone to face the back side camera, and (2) position the camera adequately. Next, while looking straight into the smartphone camera with eyes wide open, illuminate eyes with the flashlight for 4 s. Extract pupil sizes for both eyes over time from collected video, estimate key features on each eye, and report average. The PLR procedure produced complete key feature data in 94% of the cases. Redness could be produced in 97% of the cases.	Dbase	Pupil size at start of illumination
		Dcon	Pupil size at maximum contraction
		Dend	Pupil size at end of illumination
		Latency	Time to first visible pupil size reaction
		Ctime	Time to Dcon
		MCV	Max contraction velocity, the largest negative slope during illumination
		PESC	Dend-Dcon
		MCA	Dbase-Dcon
		RMCA	MCA/Dbase
		Redness	Color of the sclera in CIELAB-A color coordinate.
Non-convergence (NC)	Using voice guidance, ask the person to first look straight, and later to cross eyes, all while facing the smartphone camera. Extract horizontal iris positions over time from collected video. The NC procedure produced key feature data in 94% of the cases.	NCdiff	[Distance between eyes when looking straight] – [Distance between eyes when crossing them]
Nystagmus (NY)	Using voice guidance, ask the person to first look straight, and later to look far to the side without turning the head, all while facing the smartphone camera. Extract horizontal iris position vs. time from collected video for the eye looking to the lateral side. If horizontal nystagmus occurs it will be visible as a small peak in the graph of iris position vs. time. The NY procedure produced complete key feature data in 87% of the cases.	NYnumber	Number of peaks greater than a threshold per unit time in the trajectory of iris position over time while looking far to the side.
		NYmass	Area of the peaks identified in Peak Counts.

The position of the iris and the size of the pupil were evaluated for each frame in collected video recordings using a neural network model (AI-model). Two AI-models were tested, the one available at the start of the clinical study (the initial AI-model), and one refined model where additional data was included for training the AI-model (the refined AI-model). The primary outcome was evaluated using the initial AI-model for evaluating pupil size and key features, whereas secondary outcomes were evaluated using a refined AI-model, as detailed in Supplementary material.

Following a week of training at home where subjects used the app daily, a full day session was conducted. Prior to drug intake, each subject made three baseline tests at two light conditions and a usability questionnaire was completed.

During the drug dosing visit, data was collected in a controlled area at LUMC. Light conditions were pre-installed in a dedicated no-daylight room using smart controllable lighting equipment (IKEA) with 10 lamps (each 1,055 lumen) mounted near the ceiling in two rows forming a rectangle (Figure 1) illuminating the ceiling to produce indirect light. Light conditions were validated using a luminometer (Sekonic Flashmate L-308) in the center position, where a subject would sit on a medical recliner chair (Figure 1). The drug dosing visit starts with the collection of base line data, three tests in each light condition. Following drug administration (Table 1), ocular characteristic data was collected from subjects at hourly intervals for a duration of 5 h post administration. For the medicinal cannabis group, one extra measurement at 30 min post drug intake was performed. Eye characteristic data measurements made in the research unit were collected at two controlled light conditions, ~50 and ~ 500 lux corresponding to dim indoor lights and bright indoor lights, respectively.

Blood samples were drawn from an intravenous access line before and after drug dosing, at 6 or 7 time points to allow for pharmacokinetic analyses (Ardena Bioanalysis, Assen, Netherlands).

### Data analysis

Previct Drugs converts collected data into key features (Table 2). For PLR, key features essentially as illustrated by Robert and Charlotte (2018) were extracted. For NC, the position of the iris relative to the nose for both eyes were calculated and added to reflect the distance between irises (NCdiff). For nystagmus, the variations in horizontal position of the eye while looking to the lateral side were counted as events (PeakCount) and integrated into a mass equivalent (NYMass). The redness of sclera was estimated during the PLR procedure and the steadiness of the hand holding the smartphone was estimated during the NY procedure.

### Statistical analysis

Obtained key features were, for each subject, normalized by subtracting the sober baseline results from the ones collected under the influence of drugs. In this way, the effect of each drug is expressed as a deviation from one’s own baseline. Obtained normalized data were z-transformed using the pooled standard deviation during baseline for all subjects. The two-sided Wilcoxon rank test was used to determine if the baseline differed from the intoxicated state for the respective key features. In addition to descriptive statistics, a discriminant principal component analysis (PCA) was made using JMP-Pro 16.2.0 -statistical software (SAS Institute Inc., USA).

## Results

The primary outcome of the study was the ability of the AI-model to process the video frames into pupillograms and extract the key features of the 5 key feature families. A description of the eye-scanning procedures and the corresponding key features extracted from the acquired data are given in Table 2. The primary outcome result was that 87–97% of the acquired pupillograms produced key features. After the refining of the AI-model, the proportion was nearly identical (87–97% produced key features) but a major improvement was observed in the extracted PLR-key features for subjects with corneal arcus. In about 10% of the eye tests that failed, the use of certain medications, especially the sedative lorazepam, caused subjects to be unable to keep their eyes open throughout the test. This prevented the AI-models from accurately assessing their eyes. Details on results for the primary and all secondary outcomes are found in Supplementary materials.

All participants completed the study without serious adverse effects. In a usability questionnaire, nearly all respondents (98%) were positive or very positive toward how the app functioned (see Supplementary material). The recorded values of key features at baseline prior to drug intake, compared with those observed under the influence of one of the 4 drugs at their peak blood concentration and 5 h after drug administration is collected in Table 3. This represents the ability of each key feature to distinguish the sober condition from that induced by the test drugs. Key features related to steadiness of the hand were omitted, because no correlation between hand steadiness and drug intake could be identified (not shown).

**Table 3:** Identified differences in key features between baseline (sober) and drug conditions at peak plasma concentration and 5 h post-administration, under two ambient lighting conditions.

Key features	Benzodiazepine (Lorazepam)				Cannabinoid (Bedrocan)				Opioid (Oxycodone)				Phenethylamine (Lisdexamphetamine)			
	50 LUX		500 LUX		50 LUX		500 LUX		50 LUX		500 LUX		50 LUX		500 LUX	
	5 h	Peak	5 h	Peak	5 h	Peak	5 h	Peak	5 h	Peak	5 h	Peak	5 h	Peak	5 h	Peak
Z-NCDiff	**0.0049**	**0.0005**	**0.0029**	**0.0010**	0.3804	0.1016	0.7910	**0.0391**	0.6221	0.9697	0.2036	0.9248	0.1763	0.0830	0.1514	0.9097
Z-NYnumber	0.2324	0.1016	0.4961	0.1230	0.9102	0.4258	0.8984	0.4258	0.1763	0.1240	0.4131	0.4316	0.0977	1.0000	**0.0244**	**0.0137**
Z-NYmass	0.4922	0.1094	0.8438	0.1748	1.0000	0.4961	0.6221	0.2324	0.1963	0.2412	0.9697	0.9658	0.1763	0.5771	**0.0220**	**0.0093**
Z-Dbase	0.2334	0.0923	**0.0137**	0.1514	0.5186	0.1230	0.2334	0.9658	**0.0005**	**0.0005**	**0.0005**	**0.0010**	**0.0005**	**0.0005**	**0.0005**	**0.0005**
Z-Latency	0.5557	0.5186	0.5195	0.2578	0.6377	0.2754	0.3101	0.0674	0.9697	0.1748	0.5552	0.1230	0.6772	0.6953	0.1748	0.1812
Z-MCV	0.6772	0.2583	0.5195	0.5186	0.3013	**0.0322**	0.5322	0.2402	**0.0024**	**0.0005**	**0.0024**	**0.0010**	0.6772	0.7334	0.2334	0.2402
Z-MCVTime	0.3394	0.9097	0.3203	0.7197	0.6772	0.7002	0.6221	0.5771	0.3013	0.3394	0.0923	0.1016	0.9097	0.9658	0.6772	0.7910
Z-DCon	0.3394	0.2661	**0.0137**	0.1514	0.6772	0.6377	0.3013	0.5195	**0.0005**	**0.0005**	**0.0005**	**0.0010**	**0.0010**	**0.0010**	**0.0005**	**0.0005**
Z-Ctime	0.2402	0.3394	0.1533	**0.0269**	0.3394	**0.0303**	0.2036	0.2500	**0.0093**	0.0772	0.1099	0.1748	**0.0015**	**0.0010**	0.2661	0.4697
Z-Dend	0.4238	0.3394	**0.0029**	0.4648	0.4238	0.8984	0.1514	0.1016	**0.0005**	**0.0005**	**0.0093**	**0.0010**	**0.0010**	**0.0010**	**0.0024**	**0.0024**
Z-MCA	0.3013	0.2661	0.1016	0.3394	0.6221	**0.0029**	0.1763	0.3203	**0.0015**	**0.0005**	**0.0034**	**0.0010**	**0.0005**	**0.0137**	0.0674	0.0772
Z-Redness	0.6362	0.4238	**0.0161**	0.1763	0.0523	0.1406	0.4580	**0.0068**	0.9697	0.6221	0.2661	0.5771	0.3804	0.1475	0.8311	0.4131

The obtained key features were, for each subject, normalized by subtracting the average sober baseline results at visit 2 from the ones collected under the influence of drugs. Features with significant differences (*p* < 0.05) highlighted in bold face.

Even though data was collected using two seemingly distinct light levels (~50 lux and ~ 500 lux), the resulting ambient light level as measured in the Previct Drugs App varied significantly because study subjects made measurements at slightly different locations within the measurement area. At the lower light level, ambient light ranged from 36 to 131 lux and ranged from 187 to 512 lux for the brighter light level.

**Figure 2:**
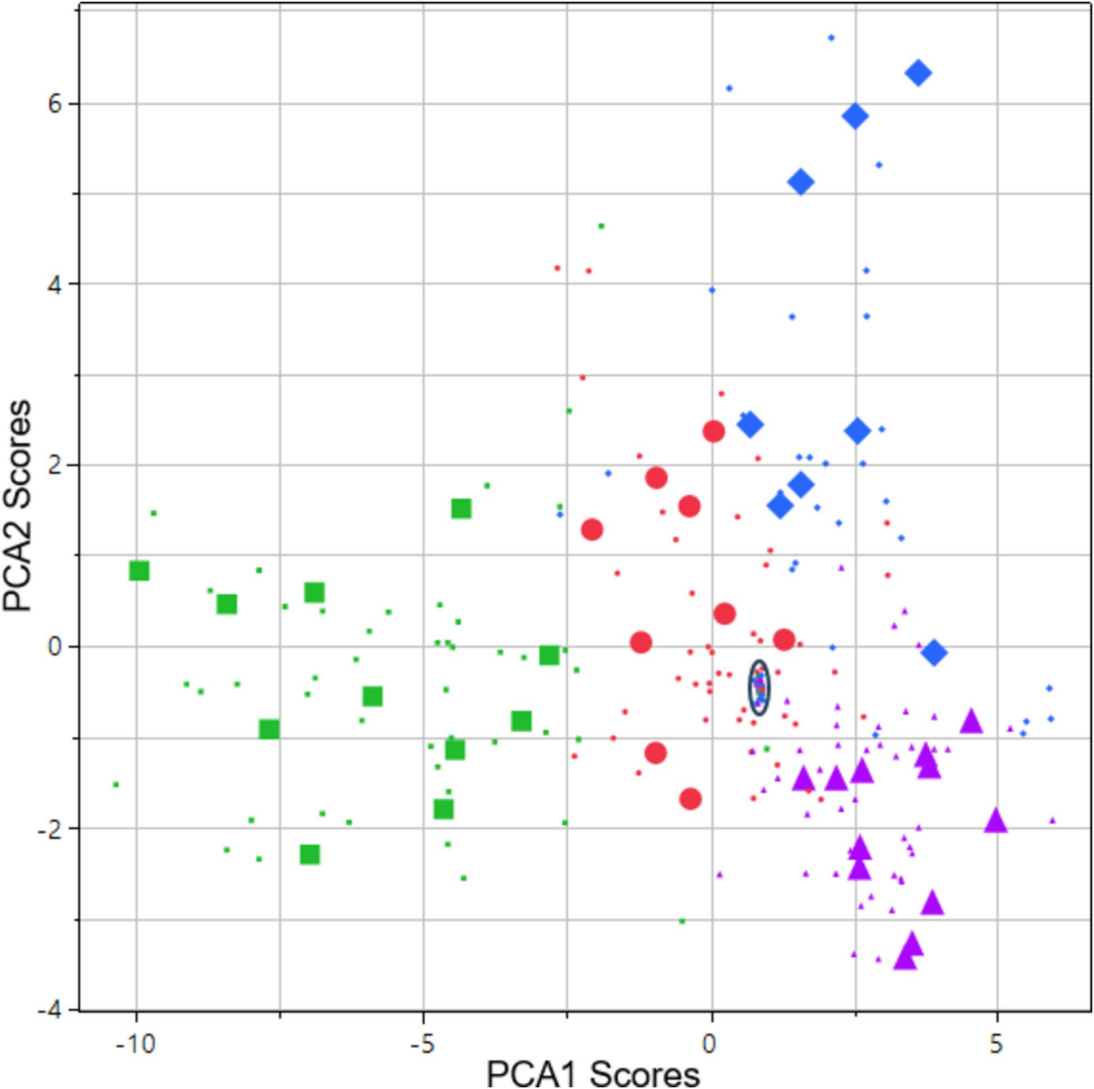
Score vector of principal components 2 plotted vs. 1. The analysis is based on all key features collected at both light conditions. Data is from 48 subjects: 12 subjects across 4 types of drug families including baseline measurements and 5–6 subsequent assessments during 1–5 h after drug administration on the day of intake. Data points originating from the time point with highest average eye reaction magnitude are shown as large size markers. In some cases, subjects failed to produce data at this time point due to intoxication, leading to fewer than 12 large size markers in some cases. Green square = opioid (oxycodone). Purple triangle = phenethylamine (lisdexamphetamine). Blue diamond = benzodiazepine (lorazepam). Red circles = cannabis (Bedrocan). Black oval = drug sober baseline (95% of collected data inside the oval).

**Figure 3:**
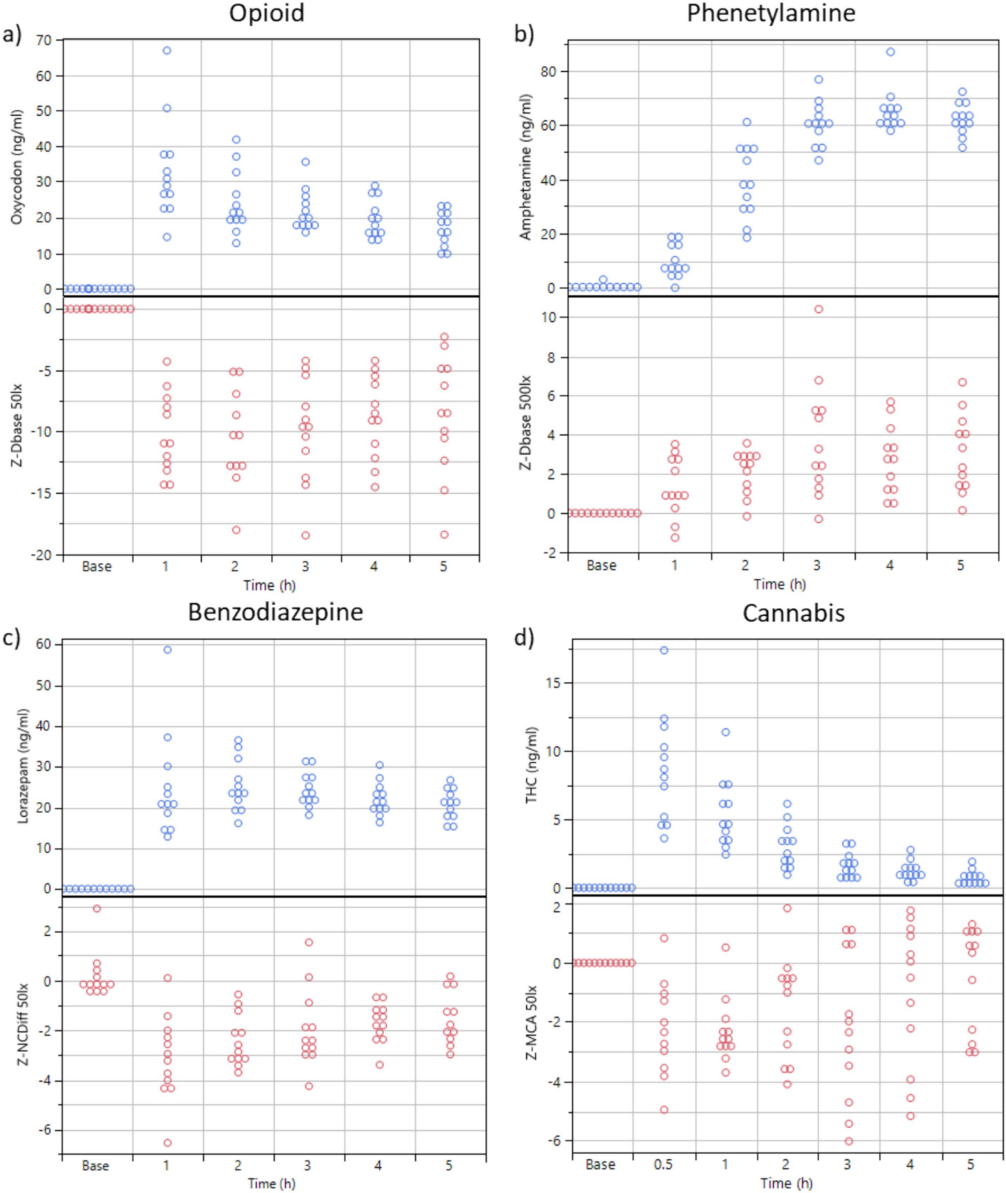
**(A,B)** Plasma concentration of oxycodone and dexamphetamine over time (*n* = 12 subjects) and the key feature values for Dbase (z-transformed, from PLR) at sober baseline and 1, 2, 3, 4, and 5 h after drug administration. Ambient illumination was ~50 lux in a and 500 Lux in **(B)**. **(C)** Blood concentration of lorazepam over time (*n* = 12 subjects) and key feature values for NCdiff (z-transformed, from NC, 50 Lux). **(D)** Blood concentration of THC over time (*n* = 12 subjects) and the key feature values for MCA (z-transformed, from PLR, 50 lux).

The first two components of a discriminant principal component analysis of all key features (transformed to z-scores) at both light conditions collected at baseline prior to drug administration and 0.5 h (cannabis only), 1, 2, 3, 4 and 5 h after drug administration are shown in Figure 2, where each drug family is encoded in a distinct color and marker type, with data collected at the time point with highest average eye reaction magnitude being shown with large size markers. The first principal component was predominantly constructed from the pupil size key features from the PLR procedure, indicating a high degree of correlation of all pupil size key features (Table 4). The second principal component was predominantly constructed from the NC and NY key features (Table 4), also indicating a high degree of correlation of NY and NC key features. Pupil reaction time (e.g., Ctime, Latency) and color of sclera represent other principal elements in eye reaction, not visible in the first two principal components (data not shown). Baseline measurements are encircled in black (Figure 2; 95% of all baseline measurements resides within the oval). All four drug types are clearly separated from the baseline measurements, in particular when assessing the time point with highest average eye reaction (large size markers). Oxycodone (green square) and lisdexamphetamine (cyan triangle) are found in the first principal component, with opposite signs. Lorazepam (blue diamond) is found in the second principal component. Data from the inhaled cannabis (red circle) are scattered in both principal components but differ from the homogenous baseline position (black). Key features were selected as representative for each principal component to allow analysis of observed effect on eyes over time. The oxycodone concentration in blood peaked at 1 h (33 ± 14 ng/mL) and declined to 17 ± 5 ng/mL at 5 h (Figure 3A, top). The z-score transformed PLR key feature Dbase at ~50 lux, which is strongly correlated with the first principal component, decreased from 0 to −10 ± 5 units at 1 h (*p* < 0.01) and stayed clearly below 0 for the full 5 h (Figure 3A, bottom).

**Table 4:** Parameter contributions to principal components 1 and 2 in the discriminant PCA.

Key feature; light condition	PC1	PC2
Z-DbaseBinNorm;50lux	0.96	0.02
Z-DbaseBinNorm;500lux	0.95	0.04
Z-MCABinNorm;50lux	0.94	0.06
Z-DConBinNorm;50lux	0.88	0.07
Z-MCABinNorm;500lux	0.87	0.08
Z-DConBinNorm;500lux	0.86	0.05
Z-MCVBinNorm;50lux	0.86	−0.01
Z-RMCABinNorm;500lux	0.85	−0.01
Z-MCVBinNorm;500lux	0.84	−0.01
Z-RMCABinNorm;50lux	0.83	−0.04
Z-DendBinNorm;500lux	0.80	0.04
Z-CtimeBinNorm;50lux	0.51	−0.41
**IngestedLisdexamphetamine**	0.43	−0.45
Z-NYnumberNorm;50lux	−0.56	0.66
Z-NYnumberNorm;500lux	−0.17	0.64
Z-NYmassNorm;50lux	0.06	0.61
**IngestedLorazepam**	0.28	0.61
Z-NYmassNorm;500lux	−0.14	0.58
Z-MCVTimeBinNorm;500lux	−0.24	0.22
Z-CtimeBinNorm;500lux	−0.19	0.42
**IngestedOxycodone**	−0.83	−0.11
Z-NCDiffNorm;500lux	−0.20	−0.55
Z-NCDiffNorm;50lux	−0.23	−0.62
Z-RednessBinNorm;500lux	0.10	0.23
Z-RednessBinNorm;50lux	−0.29	0.33
**IngestedBedrocan (THC)**	0.09	0.23
Z-PESCBinNorm;500lux	0.20	0.20
Z-LatencyBinNorm;50lux	−0.05	0.28
Z-LatencyBinNorm;500lux	0.01	0.32
Z-MCVTimeBinNorm;50lux	−0.27	0.22
Z-PESCBinNorm;50lux	−0.64	−0.10

Discriminant parameters are indicated in bold face.

The dexamphetamine concentration in blood slowly increased for 3 h and peaked at 4 h (64 ± 8 ng/mL) (Figure 3B). The z-score transformed PLR key feature Dbase at ~50 lux slowly increased from 0 to 4.3 ± 2.0 units at 5 h (*p* < 0.01) (Figure 3B).

**Figure 4:**
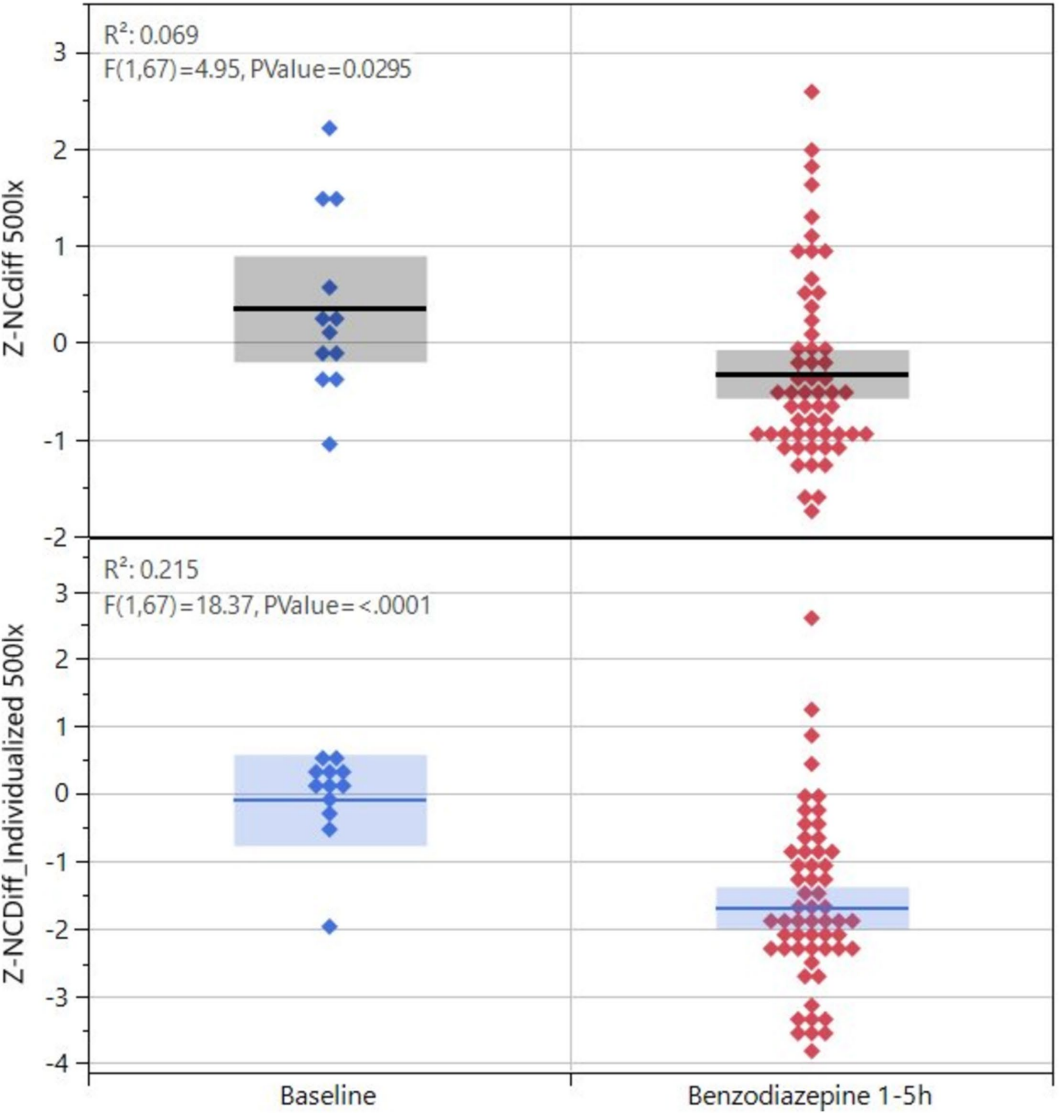
Key feature values for NCdiff for the 12 subjects exposed to lorazepam, baseline versus after drug administration, and values shown as before or after individualization.

The lorazepam concentration in blood rapidly increased to a stable level at 1, 2, and 3 h, (24 ± 13 ng/mL) and then slowly declined to 20.6 ± 3.6 ng/mL at 5 h (Figure 3C). The z-score transformed NC key feature NCDiff at ~50 lux, which is strongly correlated with the second principal component, decreased from 0 to −3.2 ± 1.7 units at 1 h (*p* < 0.01) and stayed below 0 for the full 5 h for most subjects (Figure 3C). Individual abilities to cross eyes varied widely so that the maximum ability to cross eyes for one individual could be smaller than the impaired ability to cross eyes for a different individual (Figure 4). For inhaled cannabis, the THC concentration in blood peaked in the first measurement at 0.5 h (8.6 ± 4.0 ng/mL) and rapidly decreased to 0.7 ± 0.7 ng/mL at 5 h (Figure 3D). The z-score transformed PLR key feature maximum contraction amplitude (MCA), which is strongly correlated with the first principal component, decreased from 0 to −2.2 ± 2 units at 0.5–1 h (*p* < 0.01) and after that gradually approached baseline level (Figure 3D). The reaction of the eye was delayed compared to the blood concentration trajectory. Four subjects had markedly lower blood concentration of THC (~5 ng/mL). MCA was higher in absolute values at ~50 lux compared to ~500 lux.

**Figure 5:**
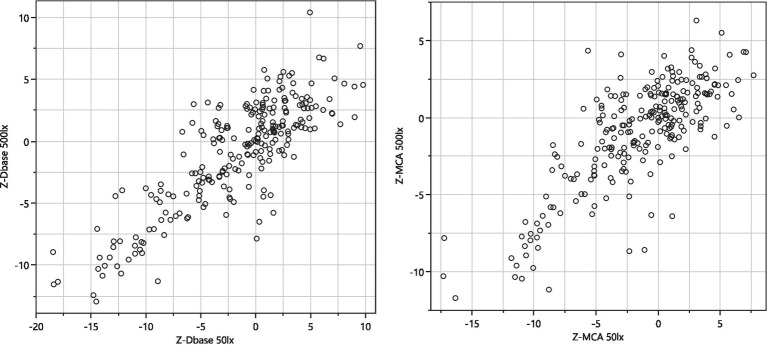
Correlation of selected key feature z-transformed values (Dbase, left planel and MCA, right panel) collected at ~50 lux and ~500 lux.

**Figure 6:**
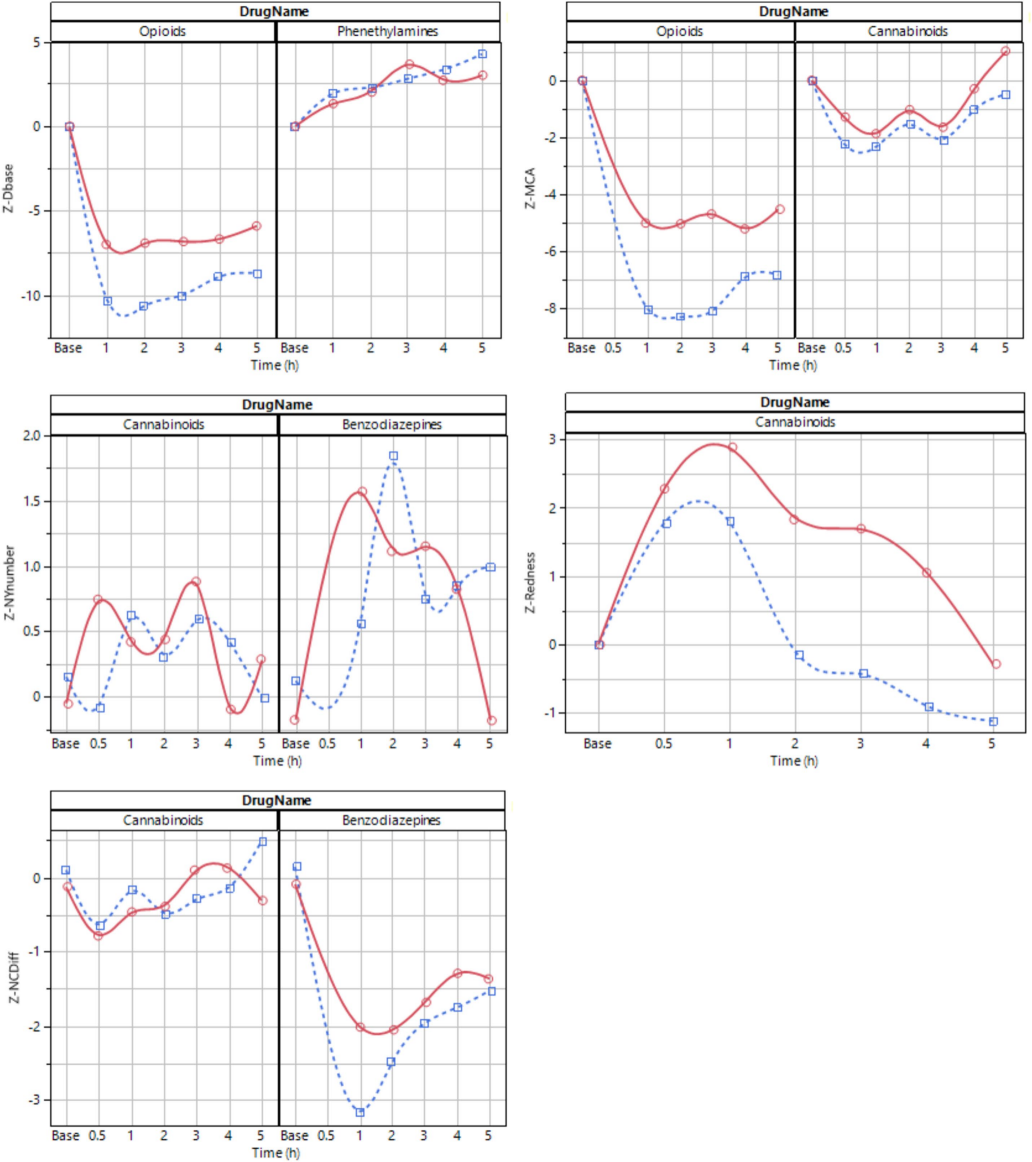
Average time series profile for selected key features at ~50 lux (blue dotted) and ~ 500 lux (red solid). The line is a spline fit provided to improve interpretability.

The PLR procedure produced results that were dependent on the ambient light during measurement. The experimental design was such that each test series started with measurements in ~50 lux ambient light, followed by a change to ~500 lux and 10 min adjustment time to let the eyes of the subject accommodate to the change in ambient light followed by a test series in ~500 lux. Hence there is a time difference of up to 20–30 min between the two light conditions. Although the eye reactions demonstrate similarity and correlation (Figure 5), data obtained under one lighting condition cannot be extrapolated to another, given the differences in the time points at which measurements were conducted. The light dependency and the discrimination power (baseline vs. drug effect) differ between drug classes (Figure 6). The PLR key features have a larger discrimination power at ~50 lux compared with ~500 lux, because at brighter light the dynamic range is compressed due to the initial smaller pupil size. This has a particular impact on opioid discrimination due to the miotic effects of opioids (Knaggs et al., 2004). For NY and NC related key features, the light dependence is non-significant (Table 3). Regarding the key feature of eye redness with bright light conditions, cannabis exhibited larger z-score redness values (Table 3).

The magnitude of differences between baseline and drug effect appeared different for different drug types, with the magnitude of changes declining as follows: oxycodone > dexamphetamine > lorazepam > cannabis (Figure 6). When ranking information content, the PLR procedure had most explanatory power for the drugs used in this study. Nystagmus had the least explanatory power, predominantly due to noise in key feature values.

## Discussion

We evaluated the eye reactions of volunteers under the influence of one of four different drugs using a smartphone app and were able to detect features that correlate with drug administration. All four drugs can be distinguished from sober condition at therapeutic dosing in controlled ambient light conditions with statistical significance (*p* < 0.01 for all four drugs). Our study also confirms recent findings (Månflod et al., 2024) that handheld, self-conducted data collection of eye reactions using an off-the-shelf consumer grade smartphone is possible, even during intoxication in most cases.

When comparing smartphone-based data to the largely known relationship between eye function and drug consumption through historic studies using precision equipment, most findings were in agreement. Opioids are known to produce miosis (Knaggs et al., 2004; Dhingra et al., 2019; Bonfiglio et al., 2006), a result which is confirmed in our study. Phenethylamines are known to enlarge pupils (Holze et al., 2020; Dhingra et al., 2019) also as confirmed in this study. For benzodiazepines, the literature indicates that nystagmus/saccadic eye movements occur (Drummer, 2002; Dhingra et al., 2019; Safran, 1984), the pupillary-light-reflex response diminishes (Safran, 1984) and convergence decreases (Dhingra et al., 2019). Although we could confirm the occurrence of nystagmus and decreased convergence in the present study, we did not detect a diminished pupillary-light-reflex response. Decreased convergence should be assessed relative to the individual’s sober baseline, as the maximal convergence capacity varies among individuals; the impaired convergence ability in one individual may exceed the unimpaired capacity of another (Figure 4).

For cannabis, the literature is partly ambiguous and partly in disagreement with findings in our study (Robert and Charlotte, 2018). It has been suggested that almost all pupil parameters could be reliable indicators for the detection of subjects under the acute effect of cannabis (Campobasso et al., 2020). Some claim that the pupil contracts (Pickworth et al., 1997; Fant et al., 1998), others that it dilates (Dhingra et al., 2019), this in addition to findings of nystagmus (DeGregorio et al., 2021). Consistent with previous research (Campobasso et al., 2020; Sexton et al., 2000), our findings indicate that the maximum contraction amplitude (MCA) key feature diminishes, a result influenced by both the reduction of baseline pupil size before illumination (Dbase) and the increased pupil size at maximum contraction (Dcon). Hence, we argue that the described effect of cannabis on the eye response depends on the timing of measurement following drug intake, the ambient light condition, the drug dose, and if a “contracted” pupil refers to the pupil size before or after illumination. The apparent inconclusiveness of the literature in respect of the pupil contracting or dilating may hence stem from comparing measurements taken at different time points after intake and different time points in the pupillogram. It seems that if MCA is used instead of an absolute pupil size measure, results will correlate better with the ingested drug concentration. The onset of nystagmus after intake of cannabis is confirmed; our results agree with Sexton et al. (2000). Further studies are however needed to establish the impact of cannabis and its cannabinoid THC on eye reactions, because the setting in the current study did not provide sufficient blood concentration of THC in four subjects.

For eye-reactions to be visible, a sufficient drug dose is required. Oxycodone and lorazepam were administered at sufficient concentrations in this study (Figures 3, 6). For lorazepam the body seems, however, to adapt to intoxication in the manner that toward 4–5 h, the identifiable eye effects decline even though the blood concentration stayed at nearly constant levels (Figures 3, 6). The central stimulant prodrug lisdexamphetamine is a slow-release formulation of dexamphetamine, as is clearly seen in the blood concentration trajectory peaking at 4–5 h after administration (Figures 3, 6). It is possible that the eye reactions continue to increase also after the last measurement. The slow-release aspect results in a flatter and lower peak drug plasma concentration occurring later in time, which may be therapeutically beneficial but complicates drug detection with the methods utilized in this study. Cannabis is rapidly taken up with a registered peak blood THC concentration already 0.5 h after administration of the cannabis variety used in our study (Figures 3, 6). Most likely, the actual peak blood concentration occurs earlier (van de Donk et al., 2019). The eye reaction peaks later, at approximately 1 h. The effect of the drugs in our study are influenced by their pharmacokinetic and pharmacodynamic properties; possibly the effect on the eyes may be related to distinct drug effect-site compartments, i.e., occurring at different sites. Taken together, eye reactions generally follow blood drug concentrations of any specific compound, but with systematic deviations of different kinds.

The collected data set contains many correlated structures. Even though about 20 different key features were analyzed, there were only 4–5 actual eye reactions that occurred during intoxication: Pupil size, pupil reaction time, horizontal nystagmus, ability to converge eyes, and color of sclera. For example, key features related to pupil size are highly correlated, so using any one of them is often sufficient in many cases. This partly mitigates the multiple comparison problem that surfaces when many tests are made on a small set of data.

Results from the two ambient light conditions generally agreed (Figure 5) but came with different challenges. In dark conditions, the sober pupil is large—meaning that the detection of phenethylamines becomes difficult, mainly because of the large variance of sober pupil sizes under such conditions. Conversely, in bright conditions it is more difficult to detect opioids because the sober pupil is already constricted. Here the compressed dynamic range becomes the challenge. This means that any attempt to distinguish drug use from sober condition using PLR must take ambient light as input and correct for any peculiarities at the respective conditions. The non-light dependent measurement procedures (NC, NY) did not, as expected, display any clear relationship to ambient light level.

Before we can transfer our measurement system into practice, several issues need to be considered. Ambient light must be assessed for every measurement and must be included in any of the drug identification models based on PLR. Whereas this study proves the ability to distinguish sober from drug conditions, it was conducted in experimental laboratory settings on a single day. The day-to-day and the person-to-person variability must be managed, even though using oneself as baseline reduces the person-to-person variability. Interfering substances, such as prescription medicines like anticonvulsants, anticholinergics, and antihistamines (Dhingra et al., 2019) and conditions like emotional arousal (Pan et al., 2024) that also cause an effect on eye reactions must be handled. By including three different test procedures (PLR, NC, NY), the coverage of different drug types is not only extended but also made more robust. Lorazepam, lisdexamphetamine and cannabis display a significant relationship to key features from more than one procedure (Table 4). A procedure relying only on PLR would face serious challenges to detect benzodiazepines, for example. A combination of data from all three test procedures will probably bring a more robust and broad method for identifying drugs. However, performing three tests in series increases the time needed for data collection and processing time. Depending on phone quality, the time to complete all three tests is 3–6 min for a premium to medium priced phone which is still very favorable compared to the cost and time required by other test procedures.

The utilization of physiological responses as a proxy for estimating drug intake represents an implicit methodology with inherent advantages and limitations. From a legal perspective, it would be impossible to claim with certainty that a particular drug has been consumed. Any indirect finding must be confirmed with a specific chemical method where an actual molecule is detected using an analytical chemical method (e.g., HPLC/MS/MS) to be of legal value. In a care process for substance use disorder, however, the precision and accuracy of the test need not be perfect, because the consequence of a false positive result is only the care provider contacting the patient to check his or her condition. It is also known that in relapse, many individuals simply omit any testing meaning that a missed test is indicative of substance use (Zetterström et al., 2021).

At the same time, chemical tests can in some cases, such as cannabis, indicate that drugs have been ingested long after the impairment is over (DeGregorio et al., 2021; Wurz and DeGregorio, 2022). This can create complications in countries where cannabis is legal for use, but not while driving. By allowing a cognitive test like eye measurements to guide the use of chemical testing, individuals who are no longer under the influence would not be falsely accused of driving under the influence even if chemical analysis would indicate THC in the blood or urine.

In the care process for patients with substance use disorder, the self-administered test using a readily available application on a mobile phone brings new opportunities. The self-administered test has proven to be intuitive and easy to use for this patient group (Månflod et al., 2024), where AI-based voice-guidance is used to instruct the patient how to perform the test. To avoid tampering the application uses AI-based facial recognition to confirm that the right individual performs the test. A similar application has successfully been used for patients with alcohol use disorders (AUD) for remote treatment using a small Bluetooth-connected breathalyzer and an application on a mobile phone (Zetterström et al., 2021). For AUD patients the AI prediction use of the eHealth system has shown to reduce short relapses with 9% and long relapses with 18% (Wallden et al., 2024), indicating that the eHealth tool is contributing to clinical effectiveness. A similar relapse prediction for SUD is foreseeable when sufficient data is available for patients with a drug use disorder. It is known that when in relapse, tests are often omitted, leading to the omission being an indicator in itself (Zetterström et al., 2021). Relieving the patient from frequent travel to the clinic for stigmatic and invasive chemical testing and instead providing a self-administered test that can be conducted at home means that the testing frequency can increase from a few times per month to a few times per day. This also enables the healthcare provider to get more knowledge between the visits and focus on the right individuals while spending less time on traditional drug testing.

An indirect test allows monitoring for intoxication caused by different drug types, by using one set of tests. However, it’s unknown how the system responds to the simultaneous use of multiple drugs. Substance substitution and coabuse during therapy is common (Kim et al., 2021) and difficult to detect with specific chemical tests. An indirect test based on for example eye reactions makes it difficult for the patient to hide such coabuse. Also, an indirect test may be advantageous for detecting designer drugs because the development of chemical tests for them is often slow. In contrast, an indirect test relies on the eye’s response to a specific drug class rather than the exact chemical composition of the new drug, making it much faster to implement.

The clinical study described here had several advantages and limitations. The study is limited to four groups of 12 healthy volunteers that were given therapeutic doses of four different drug classes. Data acquisition was conducted in a controlled environment (one room, controlled light conditions) which is advantageous for understanding the relationship on the eye reactions but may not correspond with the real-world condition. The controlled study also provided blood concentration data for the drugs over time, helping to understand dose-effect relationships, but only within a therapeutic dosing range and up to 5 h after drug intake. Individuals with a substance use disorder often ingest higher doses than therapeutically applied (Colizzi and Bhattacharyya, 2018; Kesten et al., 2022; Bech et al., 2020; Gjerde et al., 2023), with high peak concentrations due to the specific route of administration (injection, inhalation, smoking) (Quinn et al., 1997). Such conditions are impossible to test safely in healthy volunteers.

In conclusion, we report an extensive study of how eye characteristics change due to intake of therapeutic doses of four different drug classes in healthy volunteers conducted in controlled ambient light conditions. Despite recognizing the challenges associated with real-life scenarios and drug concentrations at abuse levels, the potential for a self-administered, smartphone-based method that incorporates an implicit assessment of drug use through altered eye reactions is highly promising.

## Data Availability

The datasets presented in this study can be found in online repositories. The names of the repository/repositories and accession number(s) can be found below: clinical study data is available in the public study register (clinicaltrials.gov).
