# Supplementary material for: Eye reactions under the influence of drugs of abuse as measured by smartphones: a controlled clinical study in healthy volunteers
Source: Front Neurosci. 2025 Jan 7;18:1492246. doi: 10.3389/fnins.2024.1492246 (PMC11748063; doi:10.3389/fnins.2024.1492246)
Supplement: Supplementary file 1 [file Data_Sheet_1.DOCX]

Supplementary Material

# Study workflow

The clinical study KCClin01 (NCT05731999) included health volunteers between February 15 2023 and July 12 2023. As illustrated in Supplementary Figure 1, each enrolled subject made a first visit to the study center, and received a smartphone with the App Previct Drugs version 2.18 after all inclusion procedures were completed. Approximately one week later, after having used the App at home, the subject revisited the study center for drug administration.

# Inclusion and exclusion criteria

Inclusion Criteria: The subjects have to meet all of the following criteria to be eligible to participate the investigation:

1. Male or female healthy volunteers
2. Age 18 to 70 years
3. BMI between 18.5-30 kg/m2
4. Weight between 50-100 kg
5. Healthy as determined by the investigator or designee based on pre-study medical and surgical history and a health examination at enrolment
6. Women of childbearing potential (defined as all women who are not surgically sterile or postmenopausal for at least 1 year prior to enrollment) must have a negative urine
7. contraception from enrollment until study completion
8. No current drug usage defined as a negative urine drug test at enrollment and at visit 2
9. Able to use Previct Drugs after initial training (defined as successfully performing a test after trying maximum three times per measurement)
10. Been informed of the nature, the scope, and the relevance of the clinical investigation
11. Voluntarily agreed on participation and has duly singed the Informed Consent Form

Exclusion criteria: Subjects meeting any of the following criteria will not be permitted to participate in the investigation:

1. Participating in another clinical investigation which may affect the study outcome according to clinical judgement
2. Pregnancy or Lactating
3. Blind
4. Deaf
5. Abnormal ECG (QTc time>450 ms) at enrollment
6. Current or recent history of alcohol misuse assessed by AUDIT where ≥6 points for women or ≥8 points for men indicates a potential misuse
7. Current or history of psychiatric disorder or drug misuse assessed by M.I.N.I where the outcome will be based on clinical judgement
8. Any disease or condition that may influence pupillary reflexes based on clinical judgement
9. Undergone eye surgery that may influence pupillary reflexes based on clinical judgement
10. Ongoing treatment with medications which may interfere with eye measurements based on clinical judgement
11. Ongoing treatment with medications which may interfere with any of the medicinal products to be used
12. History or presence of allergy or serious reaction to the medicinal products to be used
13. History or presence of cardiovascular disease, e.g., arteriosclerosis, hypertension, or cor pulmonale
14. History or presence of sleep-related breath disorder
15. History or presence of gastrointestinal disease, e.g., paralytic ileus, acute abdomen, delayed gastric emptying, or chronic constipation
16. History or presence of pulmonary disease, e.g., acute pulmonary insufficiency, severe respiratory depression with hypoxia, chronic obstructive lung disease, or bronchial asthma
17. History or presence of autoimmune neuromuscular disease, e.g., myasthenia gravis
18. Not able to read or understand the local language
19. Any other condition that as judged by the investigator may make the follow-up or investigation inappropriate
20. That according to the Declaration of Helsinki is deemed unsuitable for study enrollment

# Endpoints

## Primary Endpoint

• For each medicinal product (D1-D4; oxycodone, lisdexamphetamine, lorazepam, bedrocan (THC)), the fraction of collected pupillometry data from the mobile phone application at baseline and under the influence of D1-D4, which can be transformed into pre-defined key features using native pupillogram.

## Secondary Endpoints

• For each medicinal product (D1-D4), the fraction of collected pupillometry data from the mobile phone application at baseline and under the influence of D1-D4, which can be transformed into pre-defined key features using refined pupillogram.

• For each medicinal product (D1-D4), change in key features from baseline to the LC-MS/MS verified peak concentration in plasma after administration of medicinal product at visit 2 using native or refined pupillograms.

• For each medicinal product (D1-D4), analysis and plot the correlation between key features and plasma concentration over time using native or refined pupillograms.

• For each medicinal product (D1-D4), change in key features from baseline to 5 hours after administration of medicinal product at visit 2 using native or refined pupillograms.

• For each medicinal product (D1-D4), test known combinations of key features that changes from baseline to the LC-MS/MS verified peak concentration in plasma after administration of medicinal product at visit 2 using native or refined pupillograms.

• User-friendliness of Previct Drugs evaluated by the subject at visit 2.

# Results

## Primary performance endpoint

*Table S1. Fraction of native pupillogram transformed into pre-defined key features -* ***All medicinal products pooled***

| Category | NC_Include | NY_Include | TR_Include | PLR_Include | RED_Include |
| --- | --- | --- | --- | --- | --- |
| N attempts | 1275 | 1270 | 1270 | 1306 | 1306 |
| N and (%) of successful attempts | 1198 (94%) | 1103 (87%) | 1191 (94%) | 1196 (92%) | 1268 (97%) |

*Table S2. Fraction of native pupillogram transformed into pre-defined key features -* ***Lorazepam***

| Category | NC_Include | NY_Include | TR_Include | PLR_Include | RED_Include |
| --- | --- | --- | --- | --- | --- |
| N attempts | 295 | 307 | 307 | 329 | 329 |
| N and (%) of successful attempts | 286 (97%) | 242 (79%) | 282 (92%) | 297 (90%) | 317 (96%) |

*Table S3. Fraction of native pupillogram transformed into pre-defined key features –* ***Bedrocan (THC)***

| Category | NC_Include | NY_Include | TR_Include | PLR_Include | RED_Include |
| --- | --- | --- | --- | --- | --- |
| N attempts | 339 | 333 | 333 | 321 | 321 |
| N and (%) of successful attempts | 317 (94%) | 300 (90%) | 319 (96%) | 302 (94%) | 312 (97%) |

*Table S4. Fraction of native pupillogram transformed into pre-defined key features -* ***Oxycodone***

| Category | NC_Include | NY_Include | TR_Include | PLR_Include | RED_Include |
| --- | --- | --- | --- | --- | --- |
| N attempts | 332 | 318 | 318 | 336 | 336 |
| N and (%) of successful attempts | 300 (90%) | 282 (89%) | 295 (93%) | 307 (91%) | 325 (97%) |

*Table S5. Fraction of native pupillogram transformed into pre-defined key features -* ***lisdexamphetamine***

| Category | NC_Include | NY_Include | TR_Include | PLR_Include | RED_Include |
| --- | --- | --- | --- | --- | --- |
| N attempts | 309 | 312 | 312 | 320 | 320 |
| N and (%) of successful attempts | 295 (95%) | 279 (89%) | 295 (95%) | 290 (91%) | 314 (98%) |

## Secondary performance endpoints

### The first secondary endpoint

*Table S6. Fraction of refined pupillogram transformed into pre-defined key features –* ***All medicinal products pooled***

| Category | NC_Include | NY_Include | TR_Include | PLR_Include | RED_Include |
| --- | --- | --- | --- | --- | --- |
| N attempts | 1275 | 1270 | 1270 | 1306 | 1306 |
| N and (%) of successful attempts | 1193 (94%) | 1108 (87%) | 1191 (94%) | 1223 (94%) | 1268 (97%) |

*Table S7. Fraction of refined pupillogram transformed into pre-defined key features –* ***Lorazepam***

| Category | NC_Include | NY_Include | TR_Include | PLR_Include | RED_Include |
| --- | --- | --- | --- | --- | --- |
| N attempts | 295 | 307 | 307 | 329 | 329 |
| N and (%) of successful attempts | 282 (96%) | 241 (79%) | 282 (92%) | 303 (92%) | 317 (96%) |

*Table S8. Fraction of refined pupillogram transformed into pre-defined key features –* ***Bedrocan (THC)***

| Category | NC_Include | NY_Include | TR_Include | PLR_Include | RED_Include |
| --- | --- | --- | --- | --- | --- |
| N attempts | 339 | 333 | 333 | 321 | 321 |
| N and (%) of successful attempts | 318 (94%) | 305 (92%) | 319 (96%) | 309 (96%) | 312 (97%) |

*Table S9. Fraction of refined pupillogram transformed into pre-defined key features –* ***Oxycodone***

| Category | NC_Include | NY_Include | TR_Include | PLR_Include | RED_Include |
| --- | --- | --- | --- | --- | --- |
| N attempts | 332 | 318 | 318 | 336 | 336 |
| N and (%) of successful attempts | 297 (89%) | 281 (88%) | 295 (93%) | 317 (94%) | 325 (97%) |

*Table S10. Fraction of refined pupillogram transformed into pre-defined key features –* ***lisdexamphetamine***

| Category | NC_Include | NY_Include | TR_Include | PLR_Include | RED_Include |
| --- | --- | --- | --- | --- | --- |
| N attempts | 309 | 312 | 312 | 320 | 320 |
| N and (%) of successful attempts | 296 (96%) | 281 (90%) | 295 (95%) | 294 (92%) | 314 (98%) |

### Second secondary endpoint

The change in key features is presented by using p-values for each of the 24 key features at the LC-MS/MS verified peak concentration in plasma after administration of medicinal product at visit 2.

For lorazepam, the following key features had a p-value <0.05 at peak concentration:

- Z-NCDiffNorm (at 50 and 500 Lux)
- Z-Mm5Norm (at 500 Lux)
- Z-CtimeBinNorm (at 500 Lux)

 For Bedrocan (THC), the following key features had a p-value <0.05 at peak concentration:

- Z-NCDiffNorm (at 500 Lux)
- Z-MCVBinNorm (at 50 Lux)
- Z-CtimeBinNorm (at 50 Lux)
- Z-MCABinNorm (at 50 Lux)
- Z-RMCABinNorm (at 50 Lux)
- Z-RednessBinNorm (at 500 Lux)

 For oxycodone, the following key features had a p-value <0.05 at peak concentration:

- Z-Mm10Norm (at 500 Lux)
- Z-DbaseBinNorm (at 50 and 500 Lux)
- Z-MCVBinNorm (at 50 and 500 Lux)
- Z-DconBinNorm (at 50 and 500 Lux)
- Z-DendBinNorm (at 50 and 500 Lux)
- Z-CtimeBinNorm (at 50 Lux)
- Z-MCABinNorm (at 50 and 500 Lux)
- Z-RMCABinNorm (at 50 and 500 Lux)
- Z-PESCBinNorm (at 50 Lux)

 For lisdexamphetamine, the following key features had a p-value <0.05 at peak concentration:

- Z-NynumberNorm (at 500 Lux)
- Z-NymassNorm (at 500 Lux)
- Z-DbaseBinNorm (at 50 and 500 Lux)
- Z-DconBinNorm (at 50 and 500 Lux)
- Z-CtimeBinNorm (at 50 Lux)
- Z-DendBinNorm (at 50 and 500 Lux)
- Z-MCABinNorm (at 50 Lux)

### Third secondary endpoint

The correlation between the 24 pre-specified key features and plasma concentration over time using refined pupillograms are presented in **Table S10,** containing all information and presents correlation and p-value in columns for each medicinal product type. Despite the moderate correlation strength, the significance is very high (p=0.0003-0.0000). This implies that while there is a correlation, the change in plasma concentration/key feature data is not linearly connected and/or there are differences in the pharmacokinetic/pharmacodynamic properties among individuals.

*Table S10. Correlation between key features and plasma concentration over time (all timepoints)*

| Variable^12^ | Benzodiazepines | | | | Cannabinoids | | | | Opioids | | | | Phenethylamines | | | |
| --- | --- | --- | --- | --- | --- | --- | --- | --- | --- | --- | --- | --- | --- | --- | --- | --- |
|  | 50 Lux | | 500 Lux | | 50 Lux | | 500 Lux | | 50 Lux | | 500 Lux | | 50 Lux | | 500 Lux | |
|  | Corr | P-value | Corr | P-value | Corr | P-value | Corr | P-value | Corr | P-value | Corr | P-value | Corr | P-value | Corr | P-value |
| Z-NCDiffNorm; | **-0.59** | **0.0000** | **-0.54** | **0.0000** | -0.24 | 0.0294 | -0.29 | 0.0096 | -0.17 | 0.1508 | -0.18 | 0.1248 | 0.19 | 0.1065 | 0.16 | 0.1909 |
| Z-NYnumberNorm; | 0.15 | 0.2454 | 0.23 | 0.0872 | 0.00 | 0.9775 | 0.16 | 0.1615 | 0.06 | 0.6145 | 0.12 | 0.3244 | -0.09 | 0.4619 | -0.20 | 0.0913 |
| Z-NYmassNorm; | 0.15 | 0.2487 | 0.15 | 0.2768 | 0.00 | 0.9999 | 0.14 | 0.2039 | -0.01 | 0.9193 | 0.05 | 0.6997 | -0.10 | 0.4159 | -0.28 | 0.0174 |
| Z-NYmaxAMPNorm; | -0.09 | 0.4696 | 0.03 | 0.8207 | -0.13 | 0.2319 | -0.03 | 0.7573 | -0.03 | 0.8177 | 0.00 | 0.9768 | 0.21 | 0.0803 | 0.13 | 0.2901 |
| Z-NYaverageAMPNorm; | -0.01 | 0.9140 | 0.25 | 0.0610 | -0.04 | 0.7502 | -0.02 | 0.8772 | -0.01 | 0.9571 | -0.12 | 0.3078 | 0.17 | 0.1514 | 0.11 | 0.3720 |
| Z-D1mNorm; | 0.12 | 0.3389 | 0.07 | 0.5656 | 0.25 | 0.0204 | 0.19 | 0.0843 | 0.16 | 0.1896 | 0.17 | 0.1494 | -0.11 | 0.3646 | -0.29 | 0.0130 |
| Z-D2mNorm; | 0.01 | 0.9146 | 0.03 | 0.7873 | 0.38 | 0.0004 | 0.03 | 0.8225 | 0.02 | 0.8550 | 0.23 | 0.0571 | -0.12 | 0.3265 | -0.26 | 0.0285 |
| Z-Mm5Norm; | 0.07 | 0.5961 | 0.13 | 0.3005 | 0.13 | 0.2572 | 0.07 | 0.5546 | 0.23 | 0.0567 | 0.23 | 0.0481 | -0.01 | 0.9091 | -0.07 | 0.5473 |
| Z-Mm10Norm; | 0.09 | 0.4745 | 0.20 | 0.0975 | 0.08 | 0.4929 | 0.13 | 0.2565 | 0.32 | 0.0063 | 0.28 | 0.0171 | 0.02 | 0.8915 | -0.02 | 0.8808 |
| Z-Mm30Norm; | 0.11 | 0.3642 | 0.28 | 0.0239 | 0.10 | 0.3668 | 0.11 | 0.3278 | 0.36 | 0.0018 | 0.39 | 0.0007 | -0.03 | 0.8074 | -0.02 | 0.8571 |
| Z-PaBinNorm; | -0.06 | 0.6388 | 0.01 | 0.9215 | 0.02 | 0.8912 | 0.02 | 0.8673 | 0.29 | 0.0134 | 0.06 | 0.6351 | -0.10 | 0.4077 | -0.02 | 0.8644 |
| Z-PbBinNorm; | -0.04 | 0.7689 | 0.03 | 0.7919 | 0.07 | 0.5150 | -0.08 | 0.4941 | 0.15 | 0.2186 | 0.02 | 0.8412 | -0.05 | 0.6722 | -0.08 | 0.5272 |
| Z-PcBinNorm; | 0.03 | 0.7892 | -0.03 | 0.8041 | 0.19 | 0.0931 | 0.17 | 0.1238 | 0.13 | 0.2917 | 0.03 | 0.7974 | -0.15 | 0.1963 | -0.05 | 0.6746 |
| Z-DbaseBinNorm; | 0.32 | 0.0068 | 0.22 | 0.0656 | -0.19 | 0.0882 | -0.01 | 0.9409 | **-0.60** | **0.0000** | **-0.64** | **0.0000** | **0.58** | **0.0000** | **0.55** | **0.0000** |
| Z-LatencyBinNorm; | 0.00 | 0.9791 | 0.15 | 0.2216 | -0.12 | 0.2893 | 0.14 | 0.2220 | -0.02 | 0.8875 | 0.20 | 0.0872 | -0.04 | 0.7448 | -0.06 | 0.5953 |
| Z-MCVBinNorm; | 0.23 | 0.0485 | 0.10 | 0.4192 | -0.35 | 0.0013 | -0.17 | 0.1407 | -0.66 | 0.0000 | -0.52 | 0.0000 | 0.08 | 0.4929 | 0.20 | 0.1005 |
| Z-MCVTimeBinNorm; | -0.03 | 0.7822 | 0.10 | 0.3924 | 0.13 | 0.2291 | 0.17 | 0.1218 | 0.23 | 0.0526 | 0.11 | 0.3673 | -0.04 | 0.7158 | -0.12 | 0.3318 |
| Z-DConBinNorm; | 0.21 | 0.0815 | 0.24 | 0.0435 | 0.08 | 0.4938 | 0.14 | 0.2156 | -0.50 | 0.0000 | -0.60 | 0.0000 | 0.56 | 0.0000 | 0.57 | 0.0000 |
| Z-CtimeBinNorm; | 0.34 | 0.0031 | 0.30 | 0.0126 | -0.08 | 0.4536 | 0.04 | 0.7439 | 0.01 | 0.9577 | 0.05 | 0.6820 | 0.40 | 0.0006 | 0.21 | 0.0728 |
| Z-DendBinNorm; | 0.12 | 0.3248 | 0.10 | 0.4254 | 0.08 | 0.4967 | 0.09 | 0.4159 | -0.50 | 0.0000 | -0.64 | 0.0000 | 0.47 | 0.0000 | 0.44 | 0.0001 |
| Z-MCABinNorm; | 0.34 | 0.0036 | 0.15 | 0.2138 | **-0.39** | **0.0003** | -0.13 | 0.2634 | -0.58 | 0.0000 | -0.53 | 0.0000 | 0.43 | 0.0002 | 0.37 | 0.0012 |
| Z-RMCABinNorm; | 0.31 | 0.0089 | 0.09 | 0.4794 | **-0.49** | **0.0000** | -0.21 | 0.0624 | -0.62 | 0.0000 | -0.55 | 0.0000 | 0.19 | 0.1042 | 0.22 | 0.0633 |
| Z-PESCBinNorm; | -0.22 | 0.0579 | -0.22 | 0.0652 | -0.01 | 0.9505 | -0.06 | 0.6246 | 0.26 | 0.0313 | -0.20 | 0.1027 | -0.18 | 0.1214 | -0.08 | 0.5112 |
| Z-RednessBinNorm; | 0.05 | 0.6531 | 0.14 | 0.2391 | **0.51** | **0.0000** | **0.39** | **0.0003** | -0.12 | 0.3099 | -0.20 | 0.0995 | -0.15 | 0.2212 | 0.19 | 0.1184 |

### Fourth secondary endpoint

The change is presented by using p-values for each of the 24 key features at 5h after administration of medicinal product at visit 2, see **Table 3** in manuscript**.**

 For lorazepam, the following key features had a p-value <0.05 at 5h post drug intake:

- Z-NCDiffNorm (at 50 and 500 Lux)
- Z-DbaseBinNorm (at 500 Lux)
- Z-DConBinNorm (at 500 Lux)
- Z-DendBinNorm (at 500 Lux)
- Z-RednessBinNorm (at 500 Lux)

For bedrocan (THC), the following key features had a p-value <0.05 at 5h post drug intake:

- Z-NYmaxAMPNorm (at 50 Lux)
- Z-NYaverageAMPNorm (at 50 Lux)

For oxycodone, the following key features had a p-value <0.05 at 5h post drug intake:

- Z-DbaseBinNorm (at 50 and 500 Lux)
- Z-MCVBinNorm (at 50 and 500 Lux)
- Z-DConBinNorm (at 50 and 500 Lux)
- Z-DendBinNorm (at 50 and 500 Lux)
- Z-MCABinNorm (at 50 and 500 Lux)
- Z-RMCABinNorm (at 50 and 500 Lux)
- Z-PESCBinNorm (at 50 Lux)

For lisdexamphetamine, the following key features had a p-value <0.05 at 5h post drug intake:

- Z-NYnumberNorm (at 500 Lux)
- Z-NYmassNorm (at 500 Lux)
- Z-DbaseBinNorm (at 50 and 500 Lux)
- Z-DConBinNorm (at 50 and 500 Lux)
- Z-CtimeBinNorm (at 50 Lux)
- Z-DendBinNorm (at 50 and 500 Lux)
- Z-MCABinNorm (at 50 Lux)
- Z-RMCABinNorm (at 50 Lux)

### Fifth secondary endpoint

*Table S11. Results from logistic regression testing if addition of key features improves the detection of drug use.*

| Group | Light condition Lux | Key feature | AUC | At baseline N subjects | TN | FP | At peak conc. N subjects | TP | FN |
| --- | --- | --- | --- | --- | --- | --- | --- | --- | --- |
| Lorazepam | 50 | NCdiff | 1 | 12 | 12 | 0 | 12 | 12 | 0 |
|  | 500 | NCdiff | 0.88 | 12 | 10 | 2 | 12 | 11 | 1 |
|  | 500 | NCDiff-NYave | 0.88 | 12 | 11 | 1 | 11 | 10 | 1 |
| Bedrocan (THC) | 50 | MCA | 0.91 | 12 | 12 | 0 | 11 | 10 | 1 |
|  | 50 | MCA+Redness | 1 | 12 | 12 | 0 | 11 | 11 | 0 |
|  | 500 | Redness | 0.82 | 12 | 12 | 0 | 11 | 8 | 3 |
|  | 500 | Redness+NYNumber | 0.99 | 12 | 11 | 1 | 10 | 9 | 1 |
| oxycodone | 50 | Dbase | 1 | 12 | 12 | 0 | 12 | 12 | 0 |
|  | 500 | Dbase | 1 | 11 | 11 | 0 | 12 | 12 | 0 |
| lisdexamphetamine | 50 | Dbase | 1 | 12 | 12 | 0 | 12 | 12 | 0 |
|  | 500 | Dbase | 1 | 12 | 12 | 0 | 12 | 12 | 0 |

### Sixth secondary endpoint

The usability questionnaire was completed by each subject, prior to medication administration at Visit 2. The questionnaire aimed to capture the experienced user friendliness during the home testing period. The questionnaire consisted of 35 questions.

Overall, most subjects graded the Instructions For Use (IFU) “Fully understandable” (69%) and found Previct Drugs “easy to use” (98%) based on the information in the IFU. All subjects found the digital voice guidance instructions during a test easy or very easy to understand. Most subjects found it very easy (40%) or easy (52%) to perform tests with Previct Drugs. The estimated required time performing the test deviated between subjects and 12 subjects (25%) estimated the required time to <5 minutes, 22 subjects (46%) to 5-7 minutes, and 14 (29%) to > 7 minutes. Most subjects (67%) did not receive any notifications when it was time to perform a test, and approximately 50% of subjects experienced some type of issue during the usage. Despite this, only 5 subjects (10%) required any assistance from site personnel (e.g., through phone contact) during their home testing period. Most subjects found it very easy (15%) or easy (60%) to find the condition to start the measurement. No subject found it difficult or very difficult to perform the Nystagmus test, whereas 8% of subjects found it difficult to perform the convergence (crossing eye) test, and 10% either difficult or very difficult to perform the contraction test.

# Supplementary Figure


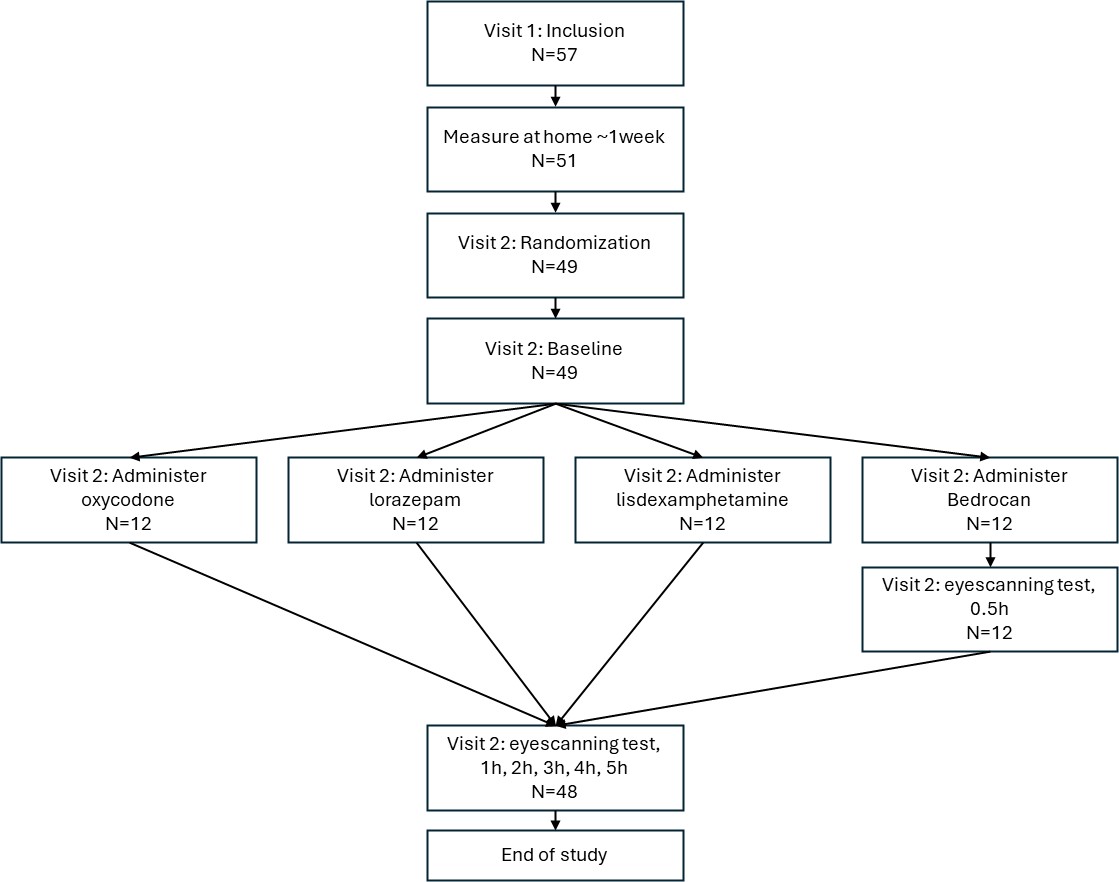


**Supplementary Figure 1.** Process chart for the clinical study. Of the 57 individuals included at visit 1, 51 proceeded with measurements at home during about one week, and 48 were administered one of the four medicinal products.
